# Systematic review for the prevention and management of falls and fear of falling in patients with Parkinson's disease

**DOI:** 10.1002/brb3.2690

**Published:** 2022-07-14

**Authors:** Wen‐Yi Liu, Tao‐Hsin Tung, Chencheng Zhang, Leiyu Shi

**Affiliations:** ^1^ Department of Health Policy and Management, Bloomberg School of Public Health Johns Hopkins University Baltimore Maryland USA; ^2^ Shanghai Bluecross Medical Science Institute Shanghai China; ^3^ Institute for Hospital Management Tsing Hua University Shenzhen Campus China; ^4^ Evidence‐based Medicine Center Taizhou Hospital of Zhejiang Province, Wenzhou Medical University Linhai Zhejiang China; ^5^ Department of Neurosurgery, Center for Functional Neurosurgery, Ruijin Hospital Shanghai Jiao Tong University School of Medicine Shanghai China

**Keywords:** falls, fear of fall, management, Parkinson's disease, prevention, systematic review

## Abstract

**Objective:**

To synthesize recent empirical evidence for the prevention and management of falls and fear of falling in patients with Parkinson's disease (PD).

**Data source:**

Database from PubMed, Cochrane Library, and EMBASE.

**Study design:**

Systematic review.

**Data collection:**

We searched the PubMed, Cochrane Library, and EMBASE databases for studies published from inception to February 27, 2021. Inclusion criteria were nonreview articles on prevention and management measures related to falls and fall prevention in Parkinson's disease patients.

**Principal findings:**

We selected 45 articles and conducted in‐depth research and discussion. According to the causes of falls in PD patients, they were divided into five directions, namely physical status, pre‐existing conditions, environment, medical care, and cognition. In the cognitive domain, we focused on the fear of falling. On the above basis, we constructed a fall prevention model, which is a tertiary prevention health care network, based on The Johns Hopkins Fall Risk Assessment Tool to provide ideas for the prevention and management of falling and fear of falling in PD patients in clinical practice

**Conclusions:**

Falls and fear of falls in patients with Parkinson's disease can be reduced by effective clinical prevention and management. Future studies are needed to explore the efficacy of treatment and prevention of falls and fear of falls.

## 1 INTRODUCTION

Parkinson's disease (PD), one of the most prevalent neurodegenerative diseases globally, causes heavy losses in social health and the economy unceasingly (GBD 2016 Parkinson's Disease Collaborators, 2018). Some experts forecasted that the number of PD patients would reach 9 million in 2030 (Dorsey & Bloem, 2018) and the neurodegenerative disease will be the dominating cause of related death by overriding cancer (Gammon, 2014). Fall is a common symptom of PD patients. Falls are often a symptom of PD, with two‐thirds of PD patients falling at least once annually (Latt et al., 2009; Paul et al., 2013), with >50% of them experiencing fall recurrence (Allen et al., 2013). Falls can increase the hospitalization rate and mortality rate (Hely et al., 1999; Soh et al., 2013; Temlett & Thompson, 2006), and PD patients with recurrent falls had decreased quality of life, even causing injury and disability (Michałowska et al., 2005; Pickering et al., 2007; Rahman et al., 2008; Soh et al., 2013). Moreover, falls may bring about fear of fall (FOF), which affects the movement of PD patients in turn and leads to the exacerbation of PD symptoms (Adkin et al., 2003; Chaudhuri et al., 2011; Rahman et al., 2011).

Some experts have suggested that assessing the joint effect of potential falls in PD patients may be useful for fall prediction (Gazibara et al., 2016a). Since our current understanding of risk factors for falls in PD patients remains poor, prevention and management strategies aiming to reduce fall occurrence have not been perfected, allowing for human and material resource loss. The combined effect of falls and FOF remains unknown. Further, ways to improve PD patients’ quality of life are arduous and lengthy.

This systematic review aims to summarize the latest evidence of falls and FOF in PD patients, outline the risk factors of falls and FOF, establish a new prevention and management model of falls and FOF, and provide ideas for clinical practice and prevention and management strategies of PD patients. It focuses on the physiological or psychological interventions in clinical practice to seek guidance and management strategies and to provide the scientific basis for future research.

## 2 METHODS

### 2.1 Literature search

**FIGURE 1:**
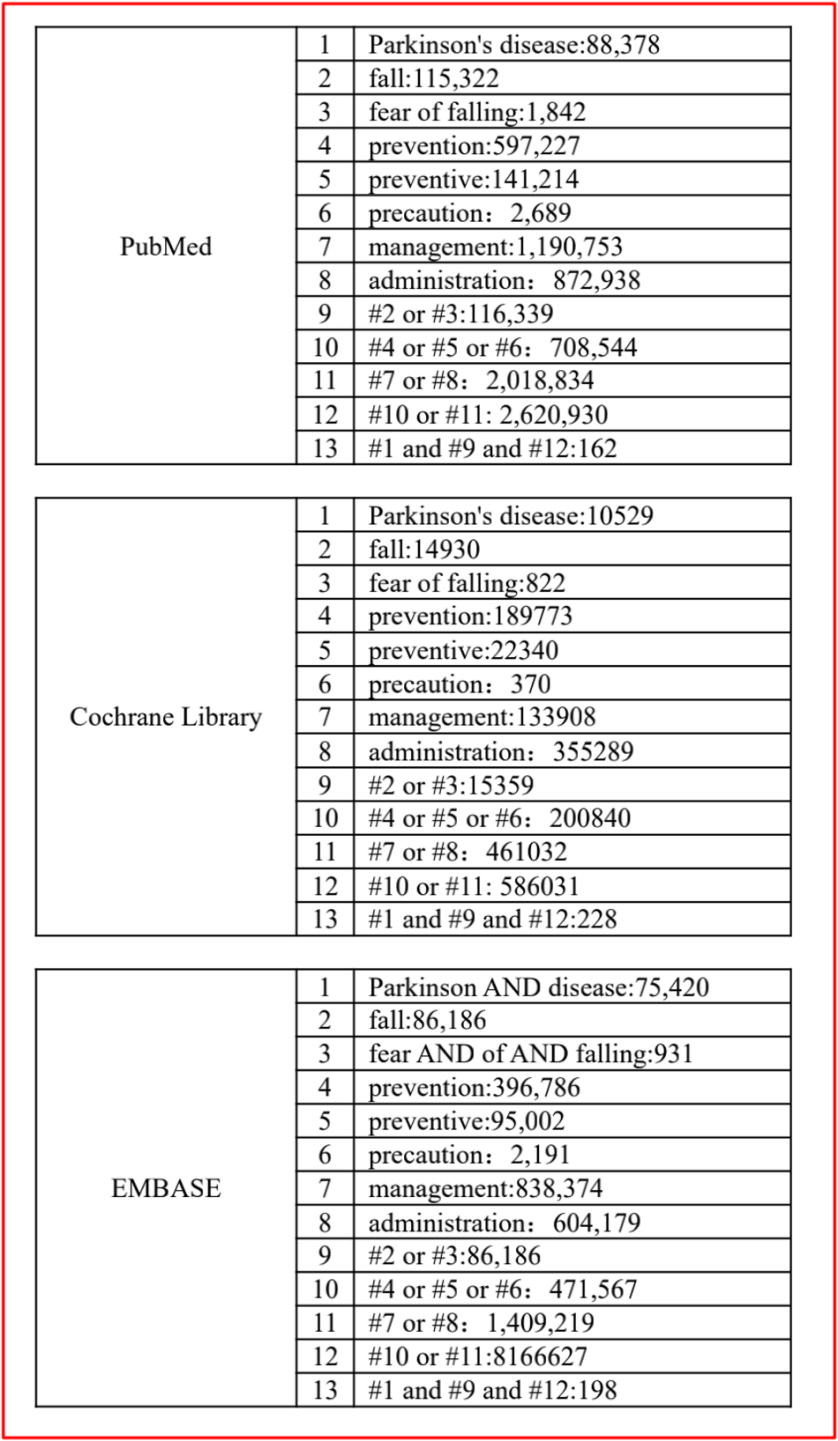
Selected stratagem of searching literature

Reports included within PubMed, Cochrane Library, and EMBASE databases published on and before February 27, 2021, were considered. The following search terms were used: fall/fear of falling, Parkinson's disease, and prevention/management, with no language limitations (Figure 1). No manual search approach was applied. The protocol of this systematic review was registered in PROSPERO (number 285709).

### 2.2 Study selection

**FIGURE 2:**
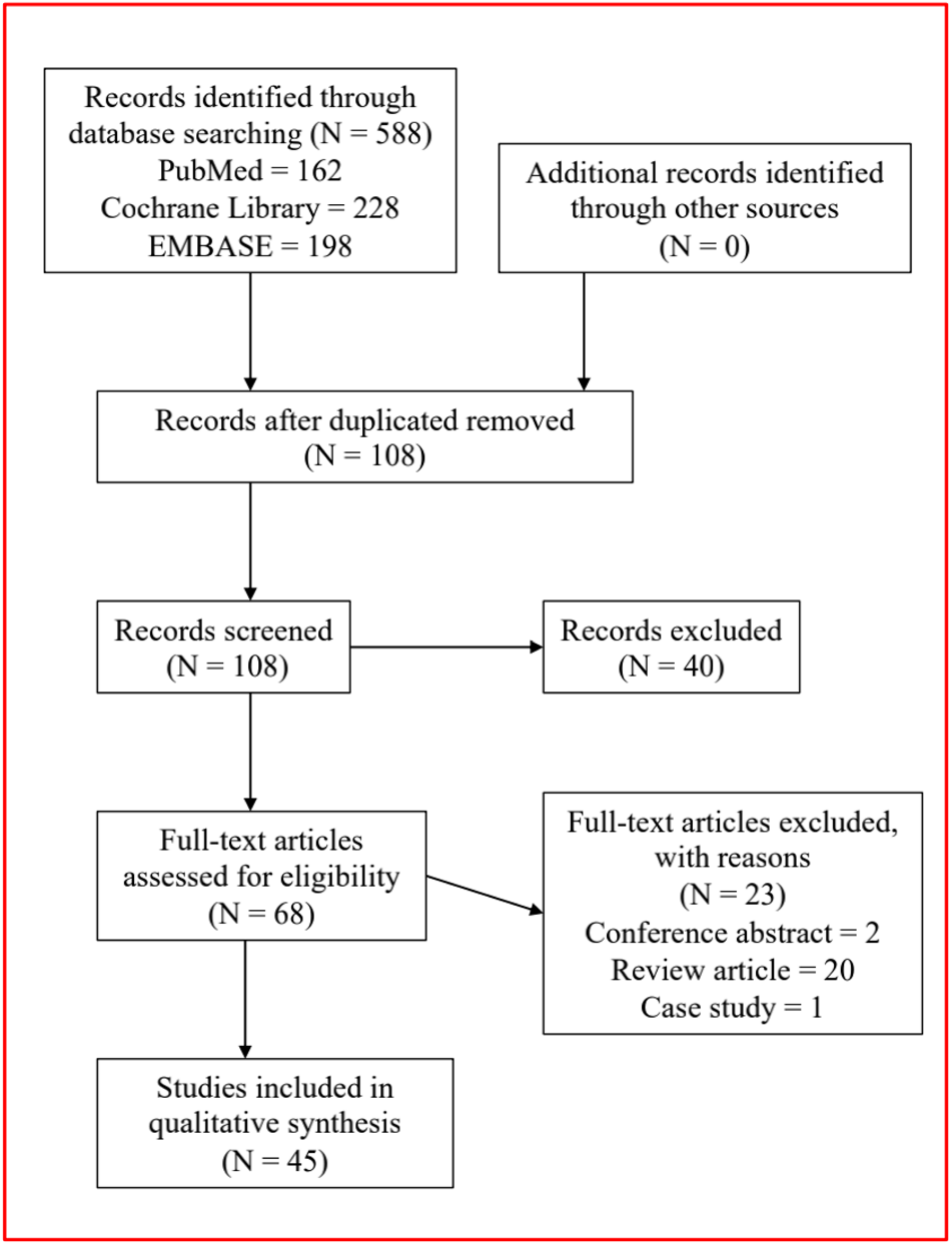
Methods of searching results and literature screening

Cross‐sectional, case‐control, prospective, and retrospective cohort studies of falls and FOF in PD were preliminarily screened. Secondary outcomes of interest included consequences of falling including injury, death, and material consumption. Reviews, case studies, and conference abstracts were excluded. Studies that summarized falls or made comparisons between falls and FOF were included. After identifying relevant articles, duplicate studies were removed. A detailed description of the search strategy and studies selected is illustrated in Figure 2. Titles and abstracts of the remaining articles were assessed. The remaining studies were further examined, with those containing information about falls, risk factors for falls, and prevention and management strategies selected. Full articles were examined to ensure relevant information was included. Two authors independently selected studies, with disagreements resolved via a discussion that included a third senior author (Figure 2).

### 2.3 Data extraction and quality assessment

We screened the full content to evaluate whether the information was potentially related. Two authors selected relevant studies independently, with disagreements resolved via discussion with a third senior author. The Newcastle–Ottawa Scale (NOS) was used to assess the quality of selected studies (Stang, 2010). Any disagreement between the two authors was also resolved by discussion with a third author. The NOS approach includes three domains (selection of study groups, comparability, and outcome assessment) to assess the quality of eligible studies. A study could be awarded up to one star for each item within the selection and outcome domains and up to two stars for comparability. If seven or more stars were awarded, we considered the study to be of high quality (Stang, 2010).

### 2.4 Data synthesis and analysis

Two broad outcome variables were considered, as follows: Risk factors for falls and FOF in PD patients (physical status, underlying health problems, environmental factors, medical care, and cognition function); and fall and FOF clinical practices (traditional measurement scales, fall prevention model, drug therapy, and surgery, and exercise). After selected studies were assessed, reasonable prevention‐management strategies for falls and FOF in PD were created.

## 3 RESULTS

### 3.1 Study selected characteristics

We obtained 162, 228, and 198 studies from the three databases, respectively. After duplicate removal, 108 studies remained. After all selection criteria were applied, 45 articles remained. Characteristics of included studies, and data regarding falls, FOF, fall‐ or FOF‐associated complications, prevention, and management are summarized in Table 1.

**TABLE 1:** Summarized results of included studies

Study, year, country, database used	Inclusion criteria	Study subjects	Outcome measures	NOS score^a^	Falls	Fear of falls	Complications of falls or fear of falls	Prevention and management
Gazibara T et al. 2014, Serbia, Neurology Clinic, Clinical Center of Serbia in Belgrade 2011–2012	Age from 22 to 83 with PD. MMSE ≥ 24, walk independently for 10 m, stand for 90 s	180 participants with PD,	Detailed interviews about falls information	S*** C** O**	Outside (57.2%), morning (53.9%), outside tripping OR: 7.90(3.21‐19.39), indoors lower extremity weakness *β*: 0.20(0.05‐0.72) and loss balance *β*: 0.19(0.05‐0.73)	NA	Soft‐tissue contusion (71.8%); fractures (12.7%)	Additional spatial visualization; using of cane; Particular prevention programs for PD at home and outside
Franzén E et al. 2016, Stockholm, Conradsson, Löfgren, Ståhle, Hagströmer, & Franzén, 2012	PD; MMSE ≥ 24	89 patients with PD; Age from 61 to 87	Structure questions, questionnaires and clinical assessments of falls; fear of falls	S** C** O**	Concerning about falling (48%)	Depression symptoms (*β* = 0.40)	NA	Focus on depressive symptoms, balance deficits, and mobility devices in rehabilitation programs of FOF
Paul SS et al. 2017, Australia, NSW Admitted Patients Data Collection 2005–2013	PD and falls	8487 fallers with PD	ICD10: S00‐T75 and T79	S*** C** O***	PD patients (2.5%); length of stay longer (*M* = 9d); indoor fall (44%)	NA	Fracture (35%); dementia (28.8%); comorbid (56.1%)	Early intervention to maintain mobility and reduce falls
Youn J et al. 2017, Korea, Movement Disorders Clinic at Samsung Medical Center, 2014–2015	PD and falls	45 participants with forward fallers, 17 with non‐forward fallers	forward PD fallers and non‐forward PD fallers	S*** C** O***	Forward falls (72.6%) non‐forward falls (27.4%). Freeze of gait is frequency in forward falls	NA	NA	Prevention strategies focusing on postural instability; Using various scales can check balance problems in PD
Friedman SM et al. 2002, USA, Health Care Financing Administration	PD; MMSE ≥ 18	2,212 participants, aged from 65 to 84	Falls and fear of falls	S*** C** O***	Who with no FOF but falls at baseline were more likely to fear at follow‐up OR: 1.97(1.46‐2.64); cut back on activities OR: 2.51(1.52‐4.14)	Who with FOF were more likely to fall than who without fear OR: 2.22(1.65‐2.98)	Female, older age, worse GHQ (*p* < 0.05)	Identify high‐risk groups; White race, female, history of stroke, sedative use, FOF were predictors; Confirm FOF is useful assessment of risk
Balash Y et al. 2005, Israel, Clinic of the Movement Disorders Unit of the Tel Aviv Sourasky Medical Center 2002	PD and falls	350 non‐demented PD patients.	falls	S*** C** O***	Advanced PD (*p* < 0.001); poor health (*p* = 0.002); duration of stance reduced (*p* < 0.001); Timed Up and Go time shorter in non‐falls (*p* < 0.001)	NA	Urinary incontinence OR: 1.95(1.17‐3.23)	Urinary incontinence can used for identify patients; Osteoporosis and treatment of osteopenia in elderly PD
Grimbergen YA et al. 2013, UK, database is not mentioned	PD and falls	74 PD patients	Falls and fear of falls	S*** C** O***	Balance confidence *β* = 0.28; Fall frequency *β* = 0.13	FOF *β* = 0.34; FOF (R^2^ = 0.53)	NA	Management to improve quality of life at prevention of falls and assessment and treatment of FOF; Prevention strategies focusing on postural instability, cognitive and emotional domains
Allcock LM et al. 2009, UK, General Practitioners outside the community screening	PD and falls	87 PD patients	Falls and fear of falls	S*** C** O**	Fall at least once (63%); falling twice or more (43.3%); accidental causes (38.9%); postural instability or dizziness (57.4%);	Continuity of attention reduced (*p* = 0.03)	Soft tissue injuries (81%)	Focus on cardiovascular and gait and balance orientated treatments and strategies to improve cognition
McKay JL et al. 2018, USA, community‐dwelling individuals 2011–2015	PD and falls	65 patients with PD and 73 normal	Falls	S*** C** O**	Falls (52%); impaired set shifting OR:1.29(1.03‐1.60); FOG (69%)	NA	NA	Set shifting may therefore be useful to include in fall risk assessments in older adults with and without PD
Gazibara T et al. 2016a, Serbia, Department of Movement Disorders, Neurology Clinic	PD and falls; walking for at least 10 m and standing for at least 90 s	120 PD patients	Falls	S** C** O**	Indoors fall (61.0%); outdoors falls (68.3%); Slipping is strongly associated with outdoor falls Indoor falls were mostly preceded by postural instability, lower extremity weakness, vertigo	NA	Fractures (4.3%) about hip fracture and redial fracture.	Using of cane; Elevating feet when crossing obstacles more than perceiving; Assess joint effect of potential falls factors; Emphasizing on balance recovery and objects in environment
Hunter H et al. 2018, UK, ICICLE‐PD	PD and falls	121 PD patients	Falls	S** C* O**	Fall diary to collect fall information	NA	Falls diary data reduced (n = 62)	Longitudinal use of falls diaries is feasible; Making personal monitoring
Hiorth YH 2014, Norway, Rogaland County, Western Norway	PD and falls	211 PD patients	Falls	S** C** O**	Disease‐specific gait and axial impairments were the major risk factors for future falls in non‐fallers at baseline	NA	NA	specific education of patients and caregivers in using compensatory strategies; Early treatment strategies of PD are important
Gazibara T et al. 2017, Serbia, Department of Movement Disorders, Neurology Clinic 2011–2012	PD and falls	120 PD patients without falls in past 6 months	Falls	S** C** O**	Fall at least one times (35%); recurrent falls (54.7%); near‐falls (93.5%)	NA	NA	engaging in tailored physical exercise may have a favorable effect on occurrence of near‐fall episodes; Focusing on balance maintenance when experiencing freezing of gait could potentially be useful in reduction of near‐falls
Kiesmann M et al. 2020, France, EVAMAR‐AGEX	PD and falls	79 PD patients	Falls within 6 months	S** C** O**	Traumatic (12%); single fallers (8%); zero fallers (34%)	NA	Hallucination (OR = 7.35); history of falls (OR = 11.78)	encouraging elder to maintain his or her cognitive abilities and physical activity focused on postural stability and posture
Dibble LE et al. 2006, USA, The University of Utah Rehabilitation and Wellness Clinic	PD and falls	45 PD patients aged 39 to 90	The individual unintentionally came to rest on the ground or other level	S*** C** O**	FRT (27.43 cm), BBS (50.20), DGI (19.92), TUG (11.67), CTUG (16.48)	NA	NA	Recode number of falls; More accurate predictive ability for falls in persons with PD
Hoskovcová M et al. 2015, Czech Republic	PD and falls UK PD brain bank criteria Without a walking aid	45 PD patients	Falls	S** C** O**	Fall one or more (60%)	NA	NA	Daylong monitoring of gait; Instrumented testing of gait in the OFF state
Moreno CM et al. 2015, Germany, database is not mentioned	PD and falls Hoehn & Yahr scale > 3	25 young PDs (12 fallers, 13 non‐fallers)	Falls	S** C** O*	Young PDs with an increased falling risk may benefit from leg‐extensors strengthening and stability training.	NA	NA	Focusing on leg‐extensors strengthening as well as on exercising the mechanisms; Developing appropriate exercise therapy
Gazibara T et al. 2016b, Serbia, the Department of Movement Disorders, Neurology Clinic, Clinical center of Serbia	PD and falls; walk independently for 10 m; statically stand for at least 90 s	120 PD patients	Falls	S** C** O***	Recurrent fallers (54.8%); outdoors falls in recurrent fallers (*p* = 0.017); slipping in single falls (36.8%); posture instability (33.0%); lower extremity weakness (*p*= 0.023)	NA	Common: soft‐tissue contusion; few: radial fracture.	Assess joint effect of potential falls factors; Enrolling in fall prevention programs; Guiding physical exercise is important
D'Cruz N et al. 2020, Belgium, the Movement Disorders Clinic of the University Hospital in Leuven	PD patients walk independently for at least 10 m	60 PD patient without freezing of gait	Falls	S** C** O**	Conversion to FOG was predicted mainly by objective and clinical measures of motor dyscontrol	NA	NA	Focusing on motor dyscontrol apparent in repetitive gait and non‐gait tasks such as finger tapping, toe tapping and stepping in place; Screening FOG conversion risk
Ashburn A et al. 2008, UK, database is not mentioned	PD; independently mobile; Living at home; falls in 12 months	142 PD patients	Falls	S*** C** O***	Home falls (80%); other falls (12%); bedroom falls (30%); loss of balance (69%); setting off too quickly after standing (11%)	NA	8 fallers fractures; 4 X‐ray; 3 need assistance.	Assisting individuals to deal with hazards cognitively and physically; Gait re‐education in PD must incorporate more than straight lines forward; Importance of cognitive; Falls diaries
Mark D et al. 2009, Australia, database is not mentioned	PD; MMSE ≥ 24; independently walk; UK PD Brain Bank criteria	130 PD patient	Falls	S*** C** O**	Falls at least once (45%); history of falls (P < 0.001); FOG (*p*= 0.004)	NA	Injurious falls (25%); 3 hip fractures, 1 radial fracture, and 1 tibial fracture	Focus on increased age, poor contrast sensitivity, slower cadence and TUG times, postural hypotension, bradykinesia, use of multiple medications, and PD‐specific factors.
Danielle PL et al. 2019, Brazil, the Movement Disorders Clinic in Fortaleza	PD patients; UK PD Brain Bank criteria	218 PD patients	Falls	S** C** O**	Disease duration, modified HY stage, SE ADL score, LED, probable sarcopenia and positive SARC‐F (SARC‐F+) were associated with falls	NA	NA	Focus on sarcopenia in the older adults; Reduced quality of life; High‐protein diet and resistance exercise training.
Silvia DD Et Al. 2020, UK, V‐TIME Study	PD; walk for 5 m; on stable medication, self‐reported 2+ falls within 6 months	282 fallers (109 older fallers, 19 MCI, 62 PD)	Falls	S** C* O**	PD falls 2 times for every 100k steps; FRA index more than other 2 groups (*p*= 0.043)	NA	NA	FRA index a preliminary but important step; V‐TIME intervention successfully reduced falls risks
Maria H et al. 2020, Sweden, Home and Health in People Ageing with PD (HHPD)	PD; mSADDE score;	151 PD patients (mean = 68±8.8 y)	Falls	S** C** O**	Fall‐related activity avoidance (16% increased); concerns about falling (*β* = 0.589);	NA	NA	Activity avoidance can be a sound strategy in hazardous circumstances; Pain is a common symptom in PD adverse outcomes
Serene SP et al. 2014, Australia, private neurology clinics	PD ;age ≥ 40, MMSE ≥ 24; walk independently	205 PD participants	Falls	S*** C* O**	Fall at least once (59%); freezing of gait (RR = 1.24); dyskinesia (RR = 1.14); stability (RR = 1.22); repeated sit to‐stand (RR = 1.19), fast walking speed (RR = 0.84); pull test (RR = 1.18)	NA	NA	Falls history probably represents a composite measure of individual risk factors; Impaired balance and cognition are important risk factors
Pattamon P et al. 2020, Thailand, Chulalongkorn Centre of Excellence for Parkinson's Disease	PD; H&Y stage 1–4; MMSE ≥ 21	305 PD patients	Having a history of at least one fall	S** C* O**	Faller (32%); recurrent fallers (19%); Model (sweep floor, reaching on tiptoes, walking in a crowded mall)	NA	NA	Determining modifiable predictors of falling; Ranking high‐risk activities as the strongest predictors of falls/recurrent falls
Chayanin F et al. 2016, Japan, Chulalongkorn Centre of Excellence for Parkinson's Disease	PD; MMSE ≥ 24;	184 PD patients and 52 normal people	Infrequent fallers; Frequent fallers	S** C** O**	HY stage higher (p < 0.001); ABC‐16 scores lower (p < 0.001); Walk on slippery sidewalks; Not holding rails on escalator; Bumped while walking in a crowd; Standing on chair to reach something	NA	NA	Activities that involved movement switching in the vertical orientation are significant predictors; Proper use of assisted devices
Tatjana G et al. 2017, Serbia, the Department of Movement Disorders, Neurology Clinic, Clinical Center of Serbia in Belgrade	PD; UK PD Brain Bank; MMSE ≥ 24; walk independently for 10 m and stand for 90 s	120 PD patient	Falls and fear of falls	S*** C** O**	NA	FES scores higher (22.9%); Taking a bath or shower is the lowest level of confidence	NA	Installing hand rails, insert bathtub chairs and/or rubber mats; Enhancing confidence
Yaroslav W et al. 2010, Germany, Movement Disorders Outpatient Departments of University Hospitals 2003–2004	PD; UK PD Brain Bank criteria	100 PD patients	Health‐economic data	S** C* O**	Falling is related with total costs (*p* < 0.05)	NA	NA	Falls is additional factors increasing PD‐related costs; Evaluating the economic burden of PD
Taylor C et al. 2017, Canada, Ambulosono walking program	PD; step‐in‐place 5 m; Non‐dementia	11 PD patients	H & Y; questionnaires	S** C** O*	Training effect (*p*= 0.047)	NA	NA	Easy and safe home‐based rehabilitation approach that may offer benefit to improving DT
Emma S et al. 2018, UK, Parkinson's UK (a UK charity) in Southampton	PD	24 PD patients involved	Sensor data of trails	S** C** O*	Recalling repeated falls (21%); fall performances: cautious (19%), unstable (5%); Rise‐to‐Walk generated most near‐falls	NA	NA	Wearable sensors can detect subtle instability and might be a useful adjunct
Nader N et al. 2019, USA, database is not mentioned	PD	18 participants with PD	Sensors of freezing of gaits.	S** C* O**	156 FOG in 18.4 min	NA	NA	Machine learning methods; Sensors detect FOG episodes, and the effects of cueing in ambient environments
Steve W P et al. 2016, UK, The North Tyneside Community Falls Prevention Service	PD; walk independently for 10 m	35,288 PD participants with 60+ age.	Falls and fear of falls	S*** C** O**	NA	Fear of falls (n = 2,448)	NA	NTCFPS was highly effective in finding individuals with falls, syncope, and dizziness symptoms; Potential clinical and health economic effect
Tara M et al. 2015, New Zealand, New Zealand Brain Research Institute database	PD; NFOGQ (having FGO); MMSE ≥ 24	21 PD participants	Falls and fear of falls	S** C** O***	Fall rate between 2 groups:1.22 (0.45‐3.26); adjusted 3.21(1.39‐9.38)	Worried about falling (17%); worried less about falling (39%);	NA	Capturing the difficulties experienced by patients in everyday life or their opinions on treatment acceptability and personal improvements
Daphne J et al. 2018, New Zealand, Leiden University Medical Center	UK PD Brain Bank clinical diagnostic criteria; age ≥ 18; stand unsupported for 20s+	30 PD patients and 30 controls	Tests of gait	S** C** O**	Walking speed, step length and stride length significant	NA	NA	It seems fair to conclude that the IWW is of added value in people with PD when assessing walking ability
Daniel SP et al. 2020, USA, database is not mentioned	PD; age ≥ 55; no other neurological disease and orthopedic injuries	16 PD patients and 14 controls	Cognitive, neuromuscular, and protective stepping	S** C** O**	Muscle onset (*p*= 0.003), step length (*p*= 0.011), latency (P < 0.001), and step width (*p*= 0.001)	NA	NA	Part of population may prioritize cognition over gait, known as a “posture second” strategy; Single‐task protective stepping can be improved through practice in people with PD
Davide C et al. 2019, Italy, NEUROFALL group 2015–2016	PD; walk 10 m independently	113 patients evolved (32 PD)	Telephone contacts for falls information	S** C** O**	Fall in the past 6 months; number of falls in the education group were evenly distributed; higher number of falls in subjects with higher level of participation	NA	NA	Education program improved ability to carry out activities and decreased participation restrictions without a concomitant increase of number of falls
Colleen GC et al. 2015; Australia, Sydney and regional and rural New South Wales	PD; age ≥ 40; walk independently; stable medication intake for 2 w; fall at least once in 1 y	231 PD patients	Falls	S*** C** O**	Exercise group compared control group (IRR = 0.73, *p*= 0.18); 69% reduction in falls in the exercise group; No significant interaction effect between fall history or physical function on rate of falls	NA	NA	Minimally supervised exercise programs aimed at reducing falls in people with PD should be implemented early in the disease process
Jennifer L et al. 2012, Australia, clinics and rehabilitation centers in Melbourne	PD; be able to walk; MMSE ≥ 24	210 participants	Mobility, activity limitations, and quality of life.	S** C** O**	Recurrent falls (64%); after intervention: 19 person falls 3–9 times,7 falls 10+ times	NA	Arthritis (44%); cancer or heart disease (23%)	Increased confidence might increase activity or risk taking and result in further falls; Strategy training and strength training can be safely implemented in a community‐based sample of people with idiopathic PD
Juliana M F et al. 2019, Brazil, database is not mentioned	PD; clock test > 4	9 PD older	After the booklets and games, creating the codes	S* C* O*	NA	NA	NA	care reduce the emotional, social and physical overload; Physiotherapy and physical activity can improve motor symptoms
Changhong Y et al. 2020, Korea, database is not mentioned	PD; H&Y stage 1–3; MMSE ≥ 24	23 participants	Test scores	S** C** O**	Step test (ES = 0.341); TUG test (ES = 0.299); AP (ES = 0.293); ML (ES = 0.299); step length (ES = 0.332), step velocity (ES = 0.301), and toe‐clearance height (ES = 0.285)	NA	NA	Exercise program may improve their overall movement; Progressive resistance exercise program
Tatjana G et al. 2015, Serbia, Department of Movement Disorders, Neurology Clinic	PD; able to walk; stand for 90 s	42 PD patients	Indoor falls and outdoor falls	S** C** O**	Indoor falls (61%); outdoor falls (68%); falls dominantly occurred in daytime; outdoors (tripping, slipping)	NA	NA	Causes of falls involved both extrinsic and intrinsic factors; Using of cane; Emphasizing on balance recovery and negotiation of objects in environment
Laura A et al. 2020, USA, database is not mentioned	PD; age ≥ 18; sMMSE ≥ 4	18 PD patients	Adverse events; FPMQ; FES‐I	S** C** O**	Falls (*n* = 10); fall decreased (elimination of recurrent fallers); 6 home falls; 4 community falls; FPMQ & FPSS had interaction between assessment time and practice	NA	NA	Learning about body awareness in yoga may have been more mindful of fall prevention; Yoga decreased the number of recurrent fallers
Natalie E et al. 2015, Australia, database is not mentioned	PD; MMSE ≥ 24; age ≥ 40; walk independently	115 PD participants	Adherence information of exercise; FES‐I; SF‐6D	S** C** O**	Adherence (72%); bodily pain (86%); less bodily pain (more SF‐6D)	NA	NA	Effective treatment of pain could therefore improve adherence to exercise
Tatjana G et al. 2016, Serbia, the Department of Movement Disorders, Neurology Clinic, Clinical Center	PD; walk for 10 m; stand for 90 s	300 PD patients	H&Y scores; UPDRS; FES; SADS; NFOG; HAMD; HAMA	S** C** O**	Recurrent fallers (19.2%); single fallers (45.2%); FES, SADS, NFOG, HAMD, HAMA positively correlated.	NA	NA	Frequent falls report less fear of falling as compared with infrequent fallers; Worse motor performance at baseline were more likely to experience recurrent falls
Kim C S et al. 2019, UK, National Health Service hospitals and clinics in UK	PD; Residuals; UK Brain Bank criteria; MMSE ≥ 24	474 PD participants	Falls; fractures; near falling; CST; GDS; FES; NFIG	S** C** O**	PDSAFE Near falling compared to control group (OR = 0.67) ;better balance (*p* = 0.026); better CST (*p*= 0.041); repeat falls (*p* = 0.111); rate of falling (*p* = 0.088)	NA	NA	Personalizing plan; Individual motivation; Integrating their training into everyday functional tasks
Emma L S et al. 2013, UK, geriatrician's clinic in UK	PD	255 PD questionnaires	Falls information (where, what, why, how, then)	S* C** O**	Single fallers(n = 19), recurrent falls(n = 86), very frequent fallers (n = 31); unfamiliar building (38%); during walking (52%); tripping (24%)	NA	Hurt (40%); immediate healthcare (16%)	Teaching them how to get up alone; Hypervigilance; Taking care to avoid trips; Sensitivity of fatigue

FOF = fear of falls; FOG = freezing of gait; PD = Parkinson's disease.

^a^ Scale domains: S selection of study groups, C comparability, O outcome assessment.

### 3.2 Classification of falls

Falls are common symptoms of PD. Fall events were defined as “some unexpected events that caused the person to unintentionally land on any lower surface such as an object, flood, or ground.” (Canning et al., 2015; Del Din et al., 2020; Foongsathaporn et al., 2016; Gazibara et al., 2016; Hoskovcová et al., 2015; Lima et al., 2020; Maki et al., 1994; Martin et al., 2015; Moreno Catalá et al., 2015; Parry et al., 2016; Paul et al., 2014) Some studies classified falls according to their frequency of occurrence, as follows: Never, few, or less; every month or year; and disabled. (Grimbergen et al., 2013) Several studies distinguished falls based on whether they occurred indoors or outdoors (Gazibara et al., 2014, 2016a, 2016b; Paul et al., 2017). Falls were also classified based on whether they occurred in a forward or non‐forward direction (Youn et al., 2017). Despite the fact that studies classified falls using different methods, all aimed to reduce falls or assess risk factors for falls.

**FIGURE 3:**
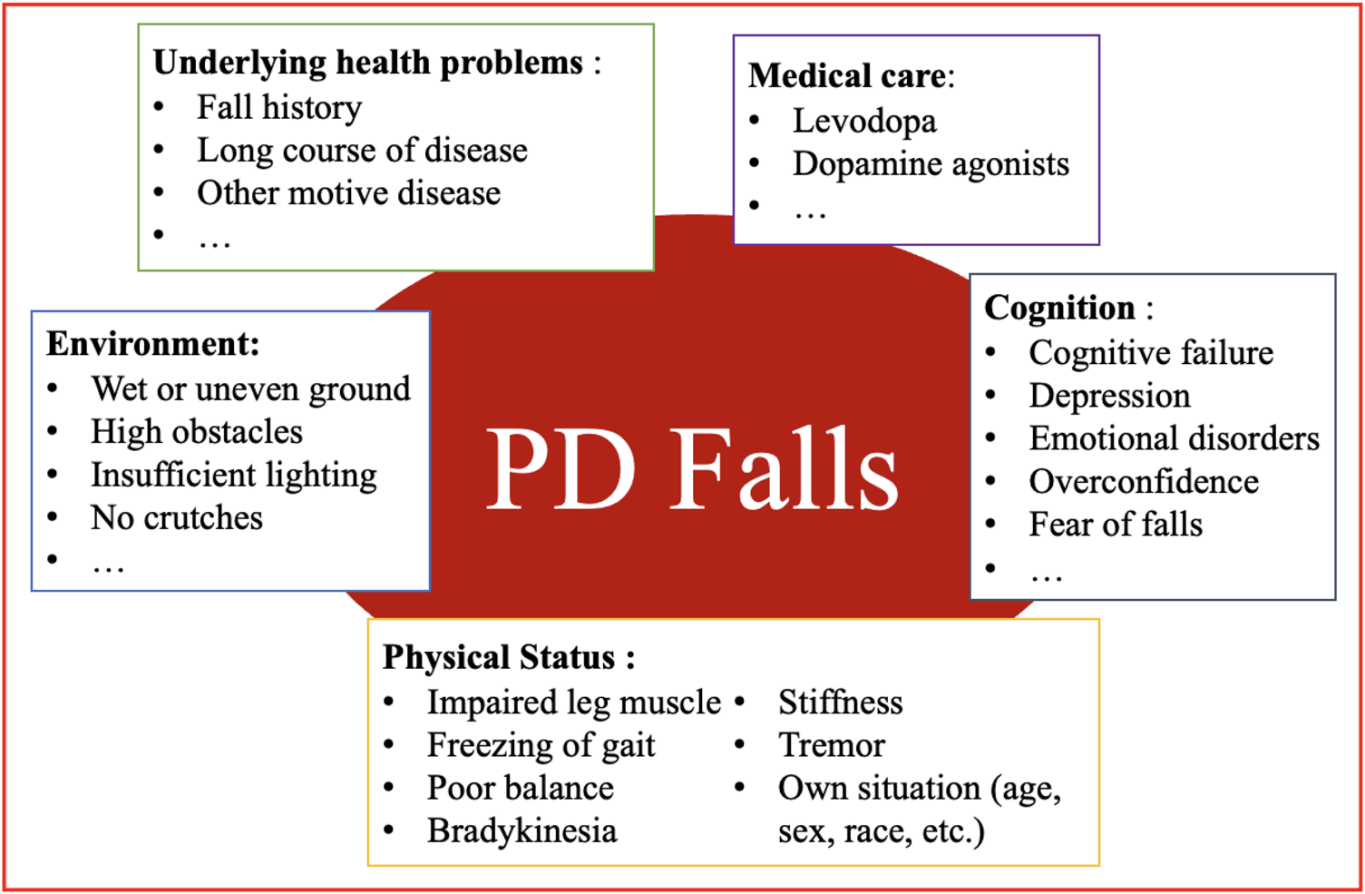
Schematic diagram of fall risk factors in patients with Parkinson's disease

The factors that may cause the fall of PD patients are summarized in Figure 3. We broke these risk factors down into five components: Physical status, pre‐existing conditions, environment, medical care, and cognition. These five parts complement each other and generally do not constitute falls in PD patients by a single factor. PD is a neurodegenerative disease, and its disease mechanism is likely to lead to falls. Therefore, we pay more attention to factors other than PD that may affect falls. Next, we will explain each of these five aspects.

#### 3.2.1 Physical status

Conclusions in different studies were inconsistent. Several predicting factors concentrated on freezing of gait (FOG), postural instability, weakness of leg muscle, and cognitive failure (Allcock et al., 2009; Ashburn et al., 2001; Bloem et al., 2001; Duncan et al., 2012; Foreman et al., 2011; Kerr et al., 2010; Latt et al., 2009; Pickering et al., 2007; Robinson et al., 2005; Wood et al., 2002). The effect of prediction was not exactly the same such as in the FOG analysis (Kerr et al., 2010; Latt et al., 2009) or in the frequency of fall analysis (Mak & Pang, 2010; Thomas et al., 2010). In clinical practice, physiological indicators such as leg weakness, postural instability, and FOG may catch physicians’ eye. Compared with the past situations, these physiological indicators can be modifiable if conducting an effective intervention. Clinicians can assess the modifiable risk factors and calculate the absolute probability of falls in the future, seeking guidance for evaluating and intervening based on indicators of motion to improve PD patients' lives (Wallace & Johansen, 2018). However, due to insufficient sample size or potential defect of studies design, these indicators' effect cannot be accurately evaluated and measured. Possible factors, such as postural instability and impaired lower limb muscle function, improved the patient's quality of life, no matter how much they improve future falls (Gazibara et al., 2014, 2016a, 2016b). Of particular concern were impaired leg muscle (Kerr et al., 2010; Latt et al., 2009), FOG (Camicioli & Majumdar, 2010; Latt et al., 2009; Paul et al., 2014), poor balance (Latt et al., 2009; Paul et al., 2014), action inconvenience (Balash et al., 2005; Cole et al., 2010; Dibble et al., 2008; Foreman et al., 2011; Kerr et al., 2010; Koller et al., 1989; Latt et al., 2009; Latt et al., 2009; Lim et al., 2008; Mak & Pang, 2010; Mak & Pang, 2009; Matinolli et al., 2011; Plotnik et al., 2011; Robinson et al., 2005; Schaafsma et al., 2003; Smulders et al., 2012). In addition, some studies have shown that the fixed attributes of people, such as age, gender, and race, have nothing to do with falls (Allcock et al., 2009; Ashburn et al., 2001; Balash et al., 2005; Latt et al., 2009; Matinolli et al., 2007; Parashos et al., 2013; Paul et al., 2013; Pickering et al., 2007), but these are all studies with small samples, and a larger sample size is needed to prove the true internal connection.

#### 3.2.2 Pre‐existing conditions

A pre‐existing poor condition is known as an underlying health problem. Age can be seen as a variable that describes health status, so old age can also be seen as a potential underlying disease. Some risk factors, include seriously injured (Allcock et al., 2009; Ashburn et al., 2001; Balash et al., 2005; Bloem et al., 2001; Camicioli & Majumdar, 2010; Contreras & Grandas, 2012; Latt et al., 2009; Lim et al., 2008; Matinolli et al., 2007; Parashos et al., 2013; Paul et al., 2013; Pickering et al., 2007), a longer course of underlying disease (Contreras & Grandas, 2012; Lim et al., 2008; Matinolli et al., 2007; Parashos et al., 2013; Paul et al., 2013; Wood et al., 2002), and, history of fall (Allcock et al., 2009; Ashburn et al., 2001; Bloem et al., 2001; Camicioli & Majumdar, 2010; Latt et al., 2009; Matinolli et al., 2011; Paul et al., 2013; Pickering et al., 2007; Wood et al., 2002), and they have been verified to be associated with falls in PD patients. For example, some researchers believe that the history of falls is a good predictor of future falls in PD patients in terms of underlying health problems (Pickering et al., 2007). Although factors related to underlying disease (including fixed factors such as age and sex) cannot be changed in the short term, they may be used to identify high‐risk groups requiring immediate preventive intervention. Further, treatment of underlying diseases may be prioritized, if appropriate.

#### 3.2.3 Environment factors

The external physical environment is an important reason for PD patients to fall, such as wet or uneven ground, high obstacles, and insufficient lighting, causing majority of the tripping or slipping (Grimbergen et al., 2004; Hely et al., 2005; Olanow et al., 2009). An observational study of fall conditions included 300 PD participants. Of the 180 people who reported falling, the conditions associated with falling included the following characteristics: Outdoors, early morning, daytime, tripping, slipping, and unsteady posture (Gazibara et al., 2014). Preventive actions that can reduce the likelihood of a fall in a complex environment include using crutches, elevating feet higher when crossing obstacles, or using armrests or pads (Gazibara et al., 2017). These external environmental factors are noteworthy in the management of PD patients, and they are also one of the factors that can most reduce the occurrence of falls.

#### 3.2.4 Medical care

In terms of medications, long‐term and high‐dose levodopa use reduced complications associated with falls (Hauser et al., 2007; Holloway et al., 2004; Parkinson Study Group, 2000; Parkinson Study Group CALM Cohort Investigators, 2009; Rascol et al., 2000). Continuous carbidopa, levodopa enteral suspension, or continuous subcutaneous apomorphine injection reduced pain levels due to complications in PD (Antonini & Nitu, 2018; Katzenschlager et al., 2018; Olanow et al., 2014). In a previously published randomized controlled trial (RCT), effects of levodopa, a dopamine agonist, and a MAO‐B inhibitor were compared, revealing that levodopa had a better activity score than the other therapeutics considered (Gray et al., 2014).

#### 3.2.5 Cognition function

Neurodegenerative symptoms of PD can lead to falling (Chaudhuri & Schapira, 2009; Chaudhuri et al., 2015, 2016). Cognitive function is particularly important in affecting falls, and good cognition can play a role in preventing falls (Chaudhuri et al., 2013). There are some factors that can be prevented and managed such as depression (Aarsland et al., 2017; Ashburn et al., 2001; Balash et al., 2005; Contreras & Grandas, 2012; Matinolli et al., 2007; Robinson et al., 2005; Wood et al., 2002), cognitive impairment (Camicioli & Majumdar, 2010; Chaudhuri et al., 2006; Ffytche et al., 2017; Latt et al., 2009; Paul et al., 2014), overconfidence (Davidsdottir et al., 2008; Gullett et al., 2013; Halperin et al., 2020; Halperin et al., 2020; Lin et al., 2014; Yakubovich et al., 2020), and FOF (Ashburn et al., 2001; Cole et al., 2010; Contreras & Grandas, 2012; Lim et al., 2008; Mak & Pang, 2009; Mak & Pang, 2009; Matinolli et al., 2007; Rahman et al., 2011; Robinson et al., 2005). These factors are grouped under cognitive classification in Figure 3. Of the most important are the effects of overconfidence and fear of falling on falls. People with PD show visual dependence because visual‐motor cues are disordered when they combine with the brain's vestibule (Bertolini et al., 2015; Halperin et al., 2020; Yakubovich et al., 2020). Perceptual overconfidence is evident, not only in vision but also in other senses such as smell (Almeida & Lebold, 2010; Azulay et al., 1999; Azulay et al., 2002; Bronstein et al., 1990; Cooke et al., 1978; Cowie et al., 2010). Visual overconfidence is associated with gait and balance and can predict the risk of falls (Curtze et al., 2016; Mak & Pang, 2009). A controlled study of 20 PD patients and 21 healthy people found that both groups had a high level of confidence in the correct target, but PD patients were more confident for the wrong reasons compared to normal people (Halperin et al., 2020).

Most falls can have minor or severe consequences, including physical and psychological injuries. One of the most important psychological harms, as opposed to overconfidence, is FOF, which is also a cognitive disorder (Adkin et al., 2003; Rahman et al., 2011). FOF has been defined as “a continuous concern about falling that contributed to individual refraining from activities” (Tinetti & Powell, 1993). The etiology and clinical symptoms of FOF show a big difference and it needs joint methods to measure. Some experts used questionnaires to estimate FOF such as the question “In general, are you afraid of falling over?” (Yardley & Smith, 2002). FOF was used to be called “basiphobia,” which is a specific type of phobia, and it manifests itself as a severe fear of standing or walking (Bhala et al., 1982; Gai et al., 2009). Although falling has been illustrated to cause FOF, FOF can also cause falls in reverse. FOF may induce falls through the change of gaits or restriction of movement (Grimbergen et al., 2013; Mak & Pang, 2009; Peto et al., 1995). Reduced activity may also lead to a reduction in the number of falls, but not the probability of falls. This suggests that FOF may indicate a decline in function due to reduced activity, leading to an increased risk of falling (Mak & Pang, 2010).

A study of the elderly in the community suggested that FOF was a dynamic process, in which the fearful stage alternated with the non‐fearful stage (Oh‐Park et al., 2011). Parkinson's disease, as a neurodegenerative disease, presents a more complex FOF than the common elderly person. Compared with healthy elderly individuals, PD patients show a higher tendency for emotional disorders (Hadjistavropoulos et al., 2011; Trollor et al., 2006), and about one‐third of PD patients suffered from anxiety disorders (Broen et al., 2016). FOF was an essential factor affecting the quality of life of PD patients (Brozova et al., 2009; Grimbergen et al., 2013). Several studies have shown that the degree of fear in recurrent fallers and frequent fallers was higher than in single fallers and low‐frequency fallers (Mak & Pang, 2010; Rahman et al., 2011; Thomas et al., 2010). A more individualized treatment approach in PD patients with FOF will bring more healthy and economic benefits to patients (Winter et al., 2010).

### 3.3 Clinical practices of falls and FOF in PD patients

#### 3.3.1 Traditional measurement scales and fall prevention model

Some traditional scales are used to predict falls and FOF, mainly including the following three scales: Consequences of falling (COF) (Yardley & Smith, 2002), falls efficacy scale (FES) (Tinetti et al., 1990), and survey of activities and fear of falling in the elderly (SAFFE) (Lachman et al., 1998; Yardley & Smith, 2002). COF rated 12 questions that described falling, with higher scores indicating greater fear of falling. FES is a self‐efficacy rating of 10 activities of daily living without falling, with higher scores indicating less confidence or a high fear of falling. And Saffe is an improved survey of activity and fear of falling in the elderly, where higher scores are associated with greater avoidance of activity. Besides, Beck Depression Inventory (BDI) (Beck et al., 1961), Beck Anxiety Inventory (BAI) (Beck et al., 1988), and assessment of quality of life scale (QOLS) (Peto et al., 1995) are measurements of mood, and can be auxiliary diagnosis methods of FOF. The scales mentioned earlier are given in Table 2.

**TABLE 2:** The detail information for 5 scales of fear of falls

Name of scales	Description	Number of questions	Score range	Scaling of score
Consequences of Falling (CoF)	Evaluating falls and how concerned you are about falls	12	12–48	Higher = Greater concern about falls
Falls Efficacy Scale (FES)	Assessing non‐hazardous activities of daily life with non‐falls	10	10–100	Higher = High fear of falls
Survey of Activities and Fear of Falling in the Elderly (SAFFE)	Assessing elderly people about fear of falls in daily activities	17	0–34	Higher = Higher avoidance of activities
Beck Depression Inventory (BDI)	Assessing depression levels	21	0–63	Higher = Greatest depression
Beck Anxiety Inventory (BAI)	Assessing anxiety levels	21	0–63	Higher = Greatest anxiety
Quality of life scale (QOLS)	Assessing quality of life	15	15–105	Higher = Greater QoL

Recently, a prospective study developed a new scale, which is an instrument to evaluate FOF in PD (Terroba‐Chambi et al., 2019). A 2‐stage and 1‐year follow‐up design validated the new scale as a self‐assessed tool for PD patients. The new scale named “Fear of Falls Scale” (FFS) contained 24 questions about daily life and clinical experience (10 questions used to evaluate the severity of motor balance ability, 10 questions used to evaluate FOF of the severity in the motor task, and 4 open questions used to supply physical activity information). It illustrated that FFS, which has simple and short duration characters, is a useful instrument to assess FOF in clinics.

**FIGURE 4:**
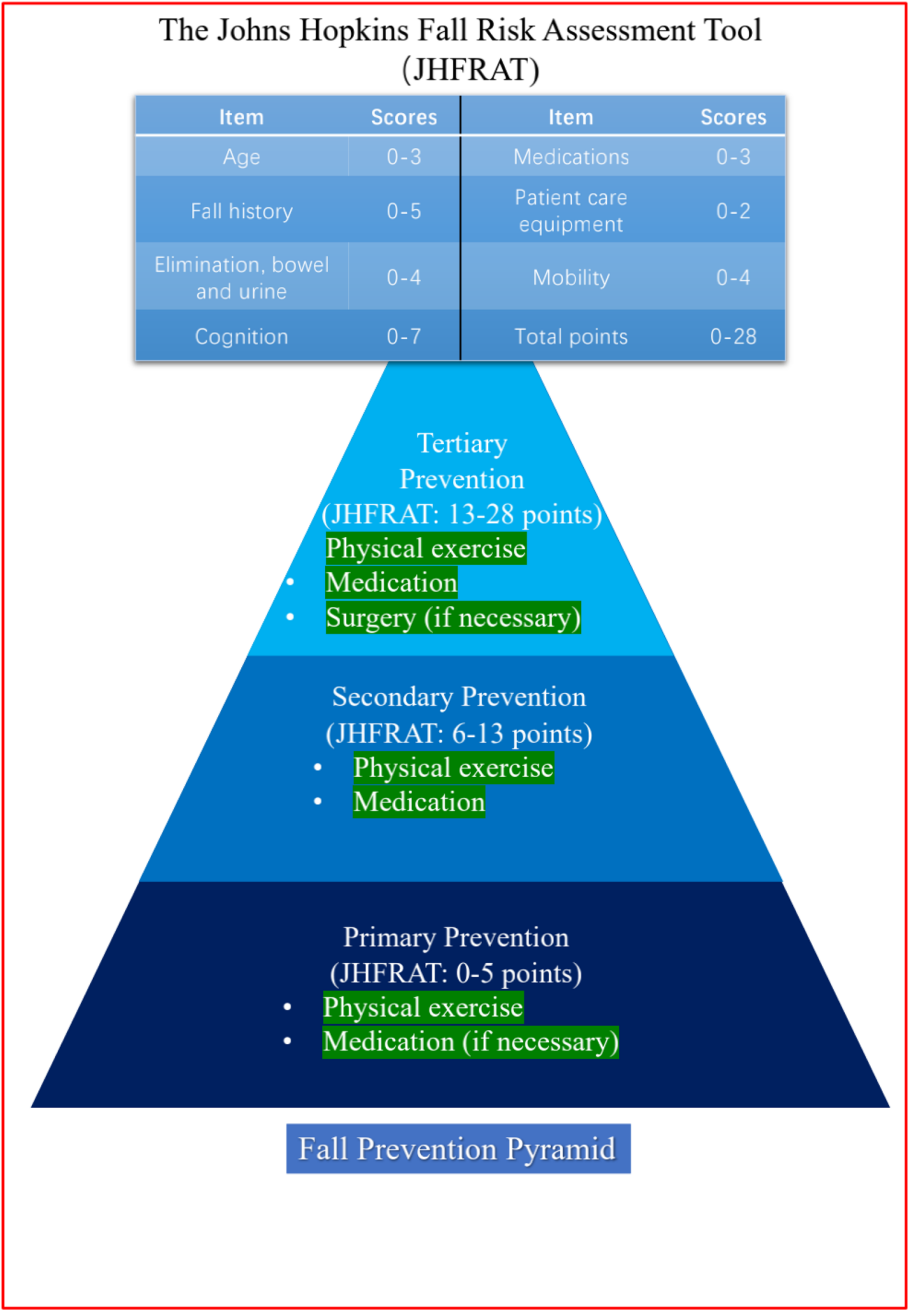
Detailed rules of The Johns Hopkins Fall Risk Assessment Tool scores and Falls Prevention Pyramid

We used The Johns Hopkins Fall Risk Assessment Tool (JHFRAT) to create the fall prevention model (details in Figure 4) to evaluate patients to determine what kind of management and treatment they receive. JHFRAT is a scale developed by Johns Hopkins University in 2003 (Poe et al., 2005). It scores patients on a scale of 0–28 based on age, history of falls, bowel movements, medication, equipment use, mobility, and cognition. Based on their scores, the JHFRAT classified patients into low‐ (0‐5 points), moderate‐ (6‐13 points), and high‐risk (>13 points) groups. JHFRAT has been shown to be a good predictor of fall risk in many studies and has been recognized by scholars worldwide (Ariza‐Zafra et al., 2020; Hur et al., 2017; Klinkenberg & Potter, 2017; Luo et al., 2020; Poe et al., 2018). We applied the risk group score to tertiary prevention in public health. In primary prevention, for a patient with a score of 0–5, we recommend a combination of medication (if necessary) and physical activity to prevent falls. In secondary prevention, we recommend that patients adhere to medication in addition to their primary prevention‐management strategy in order to improve exercise status and mental health and reduce the risk of falls and FOF. In tertiary prevention, due to the severity of the patient's condition and many complications, relevant surgical treatment should be carried out as soon as possible if the patient meets the surgical indications.

#### 3.3.2 Drug therapy and surgery methods

Drug therapy is the most common clinical treatment for patients with PD. Clinically, physicians are more likely to observe complications related to motion. Complications related to motion, such as bradykinesia, stiffness, and tremor (Postuma et al., 2015), can be treated with medication and can improve the patient's motor function and prevent falls. Dyskinesia, represented by a decrease in the velocity and amplitude of repeated movements, and stiffness, represented by an increase in auxiliary joint resistance, can be treated with levodopa (Chou et al., 2018; Hauser et al., 2000; Jankovic, 2005). Several randomized controlled trials have shown that levodopa or dopamine agonists are effective in treating motor symptoms in patients with early PD (Hauser et al., 2007; Holloway et al., 2004; Parkinson Study Group, 2000; Parkinson Study Group CALM Cohort Investigators, 2009; Rascol et al., 2000). Since the pathological feature of PD patients is insufficient secretion of dopamine, some studies have confirmed that continuous infusion of dopamine can reduce motor complications in advanced PD patients (Antonini & Nitu, 2018; Katzenschlager et al., 2018; Olanow et al., 2014). DAs currently in use include ropinirole and pramipexole, and many RCTs have been evaluated (Lieberman et al., 1998; Lieberman et al., 1997; Möller et al., 2005; Pahwa et al., 2007; Rascol et al., 1996; Schapira et al., 2011). In terms of clinical management, early PD drugs are recommended for treatment daily with 25–100 mg treatment of carbidopa‐levodopa immediate release to relieve motor symptoms and prevent falls (Freitas et al., 2016; Grosset, 2009, 2010). If the patient fluctuates during activity, the frequency of dosing may need to be increased. Adjuvant drugs can be MAO‐B inhibitors, mixed selective MAO‐B inhibitors, or ion channel inhibitors (Aradi & Hauser, 2020).

At present, there is no satisfactory drug therapy program for the clinical treatment of exercise complications. There are many individual differences in these strategies for alleviating PD symptoms. There are surgical methods of deep brain stimulation targeting at globus pallidus and subthalamic nucleus (Follett et al., 2010; Odekerken et al., 2013). Relevant RCTs show that deep brain stimulation of the subthalamic nucleus can improve motor symptoms and prevent falls (Lhommée et al., 2018). But on the other hand, subthalamic nucleus deep brain stimulation (STN‐DBS) surgery does not have an active effect on non‐motor symptoms (Amami et al., 2015; Gratwicke et al., 2018). A cohort study showed that 6 years after STN‐DBS surgery, dopamine addiction and impulse control disorders decreased, but non‐operative mental fluctuations decreased, and apathy increased (Abbes et al., 2018). In other words, surgical treatment can only improve patients' movement status and improve their physical quality to reduce falls. Moreover, these studies have not assessed the effect of mental state on falls, making it difficult to rule out a confounding effect on falls. While there has been some progress with surgery, there are still many unsolved problems in the prevention of falls, and the side effects of drugs are huge.

#### 3.3.3 Physical exercise

Although levodopa has shown promising results in the treatment of PD patients, there are serious limitations to long‐term levodopa therapy. In addition to traditional medical therapy, exercise or physical therapy is more helpful to improve the patient's motion and non‐motion status, and has benefits in increasing confidence, preventing falls, and improving quality of life (Cruise et al., 2011; Mak et al., 2017; Reynolds et al., 2016). Exercise is defined as any physical activity resulting from the expenditure of energy to contract skeletal muscles. In a large cohort, moderate‐to‐heavy exercise participants were found to have a lower risk of PD (LaHue et al., 2016), with even about two‐thirds of risk decreasing in men (Corcos et al., 2012; Mak et al., 2017). Some studies have found that physical exercise can slow down the onset and progression of PD (Cheng et al., 2016; Mak et al., 2017). Several large RCTs also found that exercise can improve symptoms of cognitive decline and bradykinesia and can effectively prevent the occurrence of falls (Corcos et al., 2012; Mak et al., 2017). Some studies can also prove that exercise intervention can alleviate the non‐motor symptoms of PD, which can relieve the mental stress of PD patients, enhance their self‐confidence, and reduce FOF (Aarsland et al., 2005; Cruise et al., 2011). Drugs do not perform well in improving the mood of PD patients, and some drugs may produce many side effects in the treatment of PD patients (Reynolds et al., 2016). This highlights the huge two‐sided benefits of exercise compared to drugs (Reynolds et al., 2016; Sacheli et al., 2018). Progressive exercise intervention for patients can restore partly behavioral ability in the physical function and prevent falls. An RCT involving 130 PD patients who were assigned treadmill‐based training therapy and physical training via biosensors showed a 55% reduction in falls within 6 months (Mirelman et al., 2016). Another study on treadmills showed that treadmills improved baroreflex sensitivity, significantly improved blood pressure, and reduced some hypotensive falls (Ganesan et al., 2014). A large study reported that low‐intensity treadmill exercise in PD patients can be as effective as medication, suggesting that aerobic exercise may improve cardiopulmonary function (Schenkman et al., 2018). Not only that, but other forms of exercise, such as Taijiquan, also have the effects of improving the movement status of patients (Song et al., 2017).

## 4 DISCUSSION

To our knowledge, this is the first review of clinical interventions and management of falls and FOF and the first presentation of a novel fall prevention theory. We focus on summarizing the existing association between falls and FOF in PD patients and attempt to summarize clinically feasible prevention and management schemes. The etiology of PD is complex, and the mechanism of this degenerative disease still needs to be studied, but this does not conflict with using existing research findings to find ways to prevent falls and the fear of falling. Because fall is a kind of subjectively unwilling but physically irresistible behavior, and the duration of falls is short, the complications of falls are extremely harmful, so it is particularly important to find a way to prevent falls. FOF is likely to occur in PD patients with or without previous falls. On the one hand, this may be the result of the neurodegenerative lesion, and on the other hand, it may be the side effect caused by PD patients' subjective perception of a different gait from the previous gait (Maki et al., 1994). FOF may lead to falls through gait changes or active restriction. If left uncontrolled, falls and FOF will interact with each other, leading to a vicious circle, and patient's quality of life will suffer unprecedented impacts. We summarized the predictive methods and influencing factors of fall and FOF, as well as the relevant methods of clinical prevention and management, which all consistently indicate that the control and management of PD are urgent.

According to our conclusion, the fall prevention model regards physical exercise as a necessary part of each stage, not only because of its good efficacy and its strong effect on improving patients' physical fitness and reducing falls, but also because it is a controllable and individualized treatment method. In primary prevention, we recommend that moderate‐intensity exercise and rehabilitation activities be used as the primary means of preventing falls and FOF, and, if necessary, take medications such as levodopa as recommended by the clinician. With the increase of JHFRAT score, we recommend that the symptoms of PD should be treated primarily, and that medication and low‐intensity exercise should be used to assist in preventing falls. Exercises are mainly low‐risk and low‐intensity exercises such as walking and tai chi. In tertiary prevention, exercise is listed as a non‐essential management item. Exercise as much as possible under the premise that patients can accept, do not cause injury and have protection, and stimulate motor nerve and cell metabolism can also reduce the risk of falling due to muscle atrophy to a certain extent (Allcock et al., 2009; Ashburn et al., 2001; Bloem et al., 2001; Duncan et al., 2012; Foreman et al., 2011; Kerr et al., 2010; Robinson et al., 2005; Wood et al., 2002).

We also looked at a state of near‐fall that could be reduced by physical exercise. A near‐fall is a transient state before a fall, which leads to two outcomes, one that leads directly to the fall, and the other that occurs shortly before the fall called “a fall initiated but arrested by support from a wall, railing, other person and so on” (Gray & Hildebrand, 2000). Near‐falls are common in people with PD. Someone will often fall if he or she loses balance and have nothing to cling to (Maidan et al., 2014). In a prospective cohort study of 120 PD patients, the association between near‐falls and falls was explored by questionnaire collection and exercise scale scores. FES was also used to assess the extent of FOF (Gazibara et al., 2017). The results showed that there was a significant association between non‐falls and falls or near‐falls in the scale scores, but no statistical association between falls and near‐falls was found. This suggests that near‐falls should be listed as a complication of PD along with falls. Medical costs for fall‐related consequences also increase as falls have more serious consequences (Pressley et al., 2003). It illustrated that taking some physical measures to reduce near‐falls or prevent falls in the state of near‐falls will bring great benefits (Gazibara et al., 2017). A multicenter randomized controlled trial also reported the risk of falls and near‐falls. After excluding the nonconforming population, 474 PD patients were randomly assigned to the experimental group for exercise and strategic intervention (PDSAFE), and for a period of 3 months for random monitoring of falls and economic evaluation (Ashburn et al., 2019). PDSAFE is a home‐based training program under the guidance of physiotherapists that includes postural control training, gait freeze reduction training, and learning feedback (Hulbert et al., 2019). This study found that the intervention significantly reduced the severity of falls and near‐falls, and the decline increased in PD patients with cognitive impairment.

The collection of fall information plays an important role in the prevention of falls. The use of retrospective self‐report may cause recall bias and may underestimate the frequency of falls (Hauer et al., 2006; Lamb et al., 2005). It is very important during intervention and management of fall prevention to correctly identify people at high risk of falls (Allen et al., 2013). A cohort study used diary data to explore its feasibility, and found that despite the high rate of loss of follow‐up, the characteristics of the people lost to follow‐up were similar to the baseline characteristics of the people who kept a diary, and the diary data was also feasible (Hunter et al., 2018). In addition, postural monitoring of patients with Parkinson's disease is crucial for the prediction and prevention of falls. With the continuous development of science and technology, more and more high‐tech equipment is being applied to the field of disease monitoring and prevention. Wearable devices, such as watches and wristbands, can already correctly identify the types of activities that occur in everyday life (Pärkkä et al., 2006). Different from traditional data collection methods, sensor devices can capture the existence of micro‐data and can use machine learning to develop relevant algorithms, which can not only understand the subtle changes in patients' falls but also objectively monitor PD symptoms and daily changes in a remote place and home (Arora et al., 2015; Lakshminarayana et al., 2014; Weiss et al., 2010). There is already a large study using wearable sensors to collect data on falls in Parkinson's patients (Silva de Lima et al., 2016). If PD patients can be screened out early and primary prevention can be carried out in time for people with suspected PD, the number of PD patients can be fundamentally reduced. For example, a large recent case‐control study with 274 participants used liquid chromatography‐mass spectrometry (LCMS) to separate and detect the presence of lipids and small molecules in the cortex of patients with PD to identify serum biomarkers of PD (Sinclair et al., 2021). The results showed that ceramides, triacylglycerol, and fatty acyl classes in PD patients decreased, while glycosphingolipid and fatty acyl metabolites increased, which is helpful for the development of skin test paper for PD patients, and has an important role in the field of public health.

Finally, the causes of falls and FOF in PD patients are complex, and epidemiological studies should be combined with basic studies to avoid various confusion caused by inaccurate data. The quaility of life of PD patients can only be improved with the participation of the whole society. Our study did not (and it was difficult to) quantify the effectiveness of prevention and management strategies in reducing falls and fear of falls, and the included literature lacked homogeneity in inclusion and exclusion criteria. We hope that there will be more research on the prevention and treatment of FOF in PD patients when they fall in the future. Although there is no data analysis in this review to support the accuracy and feasibility of the fall prevention model, the fall prevention model is established based on the existing studies and the latest theoretical basis, which can still be used as a reference measure for the prevention and management of falls in clinical practice and FOF in PD patients

## 5 CONCLUSION

Falls and FOF in PD patients can be reduced by effective clinical prevention and management. Although PD patients have a high rate of falls, FOF is common. More research is needed to explore the treatment and prevention of falls and FOF. The key is to identify these PD patients who are at high risk for falls or FOF, identify and reduce the occurrence of falls and FOF through monitoring, medication, physical exercise, and other means, in order to detect and reduce the occurrence of motor and non‐motor complications. The fall prevention model established by us expands the treatment methods of clinicians for PD patients and adopts a comprehensive prevention approach to reduce the incidence of falls and FOF. PD patients would benefit from this integrated prevention and management approach.

## CONFLICT OF INTEREST

The authors declare no potential conflicts of interest.

## AUTHOR CONTRIBUTIONS

Wen‐Yi Liu, Tao‐Hsin Tung, Chencheng Zhang, and Leiyu Shi conducted the study and drafted the manuscript. Wen‐Yi Liu and Tao‐Hsin Tung participated in the design of the study and performed data synthesis. Chencheng Zhang and Leiyu Shi conceived the study and participated in its design and coordination. All of the authors read and approved the final manuscript.

### PEER REVIEW

The peer review history for this article is available at https://publons.com/publon/10.1002/brb3.2690.

## Data Availability

All data underlying the findings are within the paper.
